# Development and validation of trigger tools in primary care: A scoping review

**DOI:** 10.1371/journal.pone.0308906

**Published:** 2025-01-02

**Authors:** Inge Dhamanti, Elida Zairina, Ida Nurhaida, Salsabila Salsabila, Fitri Yakub

**Affiliations:** 1 Department of Health Policy and Administration, Faculty of Public Health, Universitas Airlangga, Surabaya, East Java, Indonesia; 2 Center of Excellence for Patient Safety and Quality, Universitas Airlangga, Surabaya, East Java, Indonesia; 3 School of Psychology and Public Health, La Trobe University, Melbourne, VIC, Australia; 4 Department of Pharmacy Practice, Faculty of Pharmacy, Universitas Airlangga, Surabaya, East Java, Indonesia; 5 Department of Informatics, Faculty of Design and Technology, Universitas Pembangunan Jaya, Tangerang, Banten, Indonesia; 6 Malaysia-Japan International Institute of Technology, Universiti Teknologi Malaysia, Skudai, Malaysia; FMUP: Universidade do Porto Faculdade de Medicina, PORTUGAL

## Abstract

In primary care, trigger tools have been utilized to evaluate and identify patient safety events. The use of trigger tools could help clinicians and patients detect adverse events in a patient’s medical record. Due to a lack of research on the process development of trigger tools in primary care, the purpose of this scoping review is to investigate the trigger development and validation process in primary care settings. A scoping review methodology was used to map the published literature using the Joanna Briggs Methodology of performing scoping review. We considered only studies published in English in the last five years and included both qualitative and quantitative study designs. The final review included five articles. The primary care and combined primary-secondary care studies are included to gain more knowledge in the process development and validation of trigger tools. The trigger tool development process begins with clearly defining the triggers, which are then programmed into a combined computerized algorithm. The validation process was then carried out in two steps by both physician and non-physician experts for content and concurrent validity. The sensitivity, specificity, and positive predictive value (PPV) of the final algorithm were critical in determining the validity of each trigger. This study provided a comprehensive guide to developing trigger tools, emphasizing the importance of precisely defining triggers through a thorough literature review and dual validation process. There were similarities in the development and validation of trigger tools across primary care and hospital settings, allowing primary care to learn from hospital settings.

## Introduction

In primary care, trigger tools have been used to evaluate and identify patient safety events [1, 2]. It is a method of conducting a structured review of a patient’s electronic medical record in order to identify specific indicators associated with adverse events and patient harm. The Institute for Healthcare Improvement (IHI) has developed the most well-known indicators or triggers [1]. This tool has been described as extremely useful for detecting any adverse events, whether preventable or not, in outpatient and primary care settings [3]. Detected preventable AEs using trigger tools may be problematic, but they were critical opportunities for many safety interventions and were easily accepted by providers due to their intuitive appeal as quality improvement targets [4]. The trigger tool has steadily assisted in the identification of adverse drug reactions, surgical complications, and other potentially avoidable harm [5]. Furthermore, it can be used for a variety of purposes, such as providing a practice alert in order to improve the quality and safety of care. Trigger tools can be used not only to detect errors or adverse events, but also to identify the progression of chronic disease from a patient’s EMR. It serves as a ‘backstop’ for clinicians viewing results and encourages reflection if clinical review or referral is indicated [6]. The trigger tools encourage clinicians to conduct a chart review and examination.

The use of trigger tools is advantageous to both clinicians and patients. It assists clinicians by reducing the number of work-hours required to review processes in detecting each adverse event from a patient’s electronic medical record in primary care [7]. Trigger tools enable physicians to take proactive precautions against patient safety events based on triggers detected in the patient’s electronic medical record [8]. Trigger tools benefit patients by preventing harmful events, eliminating complications in medication-related errors, and achieving a safe higher level of care [4].

The trigger tool development process begins with the development the triggers and the scanning of patient electronic health records (EHR) data for clinical and diagnostic clues in order to identify hazards or risks, which are then validated or evaluated [8, 9]. An expert panel evaluated the appropriateness of the set of triggers based on hospital settings after conducting a detailed literature review and study design [10]. An algorithm were also developed due to the automated identification process of information within an EHR that signal the potential error or an adverse event through the pilot hospital’s health information technology system [5].

The trigger tools must be validated or evaluated after the development process is completed. The validation process was carried out by developing the definition of each trigger and selecting random medical charts that had been identified as positive to be evaluated by experts for the presence of triggers [11]. The validation process includes a double review by physicians or other experts to assess the sensitivity and specificity of the triggers used [12]. There have only been a few previous studies on developed trigger tools for primary care settings [9].

The studies on the development and validation of trigger tools discussed above show that the majority of them are conducted in hospitals [5, 8, 10–12] rather than primary care [9]. It has been demonstrated that there is still a dearth of recent and focused studies on trigger development and validation in primary care settings. A specific trigger tool development and validation in the primary care setting has yet to be investigated. As a result, the purpose of this study is to investigate the trigger development and validation process in primary care settings.

## Material and methods

### Inclusion criteria

The methodology used in this review adheres to the Joanna Briggs Institute (JBI) methodology guidelines, which outline a comprehensive scoping review approach [13]. Our investigation is centered on elucidating the difficulties associated with the identification, development, and validation of trigger tools in primary care settings. We included both qualitative and quantitative study designs within our scope to ensure a thorough analysis. A specific criterion for inclusion in our review was that studies be published in English, and we restricted our scope to research conducted between 2016 and 2023. The meticulously devised protocol for this scoping review was guided by the Preferred Reporting Items for Systematic reviews and Meta-Analyses extension for Scoping Reviews or PRISMA-ScR; ensuring a structured and transparent methodology [14].

### Search strategy

On March 12^th^, 2022, we conducted initial research using all identified keywords and index terms in the electronic databases PubMed and CINAHL. Due to a lack of literature on the subject, we added two more electronic databases on March 15th: ProQuest and Scopus. We updated the search on October 9^th^, 2023. The search strategy for all databases was the same as shown in the PRISMA Flowchart (S1 Fig PRISMA flowchart). In the search, keywords with Boolean operators ("OR" and "AND") related to process development and trigger tool validation were used (S1 Table. Search Terms) [13]. These keywords were used in conjunction with a comprehensive list of key term variations. The reference lists of all identified reports and articles were also searched. We did not search the gray literature because we are only interested in studies published in peer-reviewed journals that are based on scientific methods that use evidence to develop conclusions.

### Eligibility criteria and data selection

**Fig 1:**
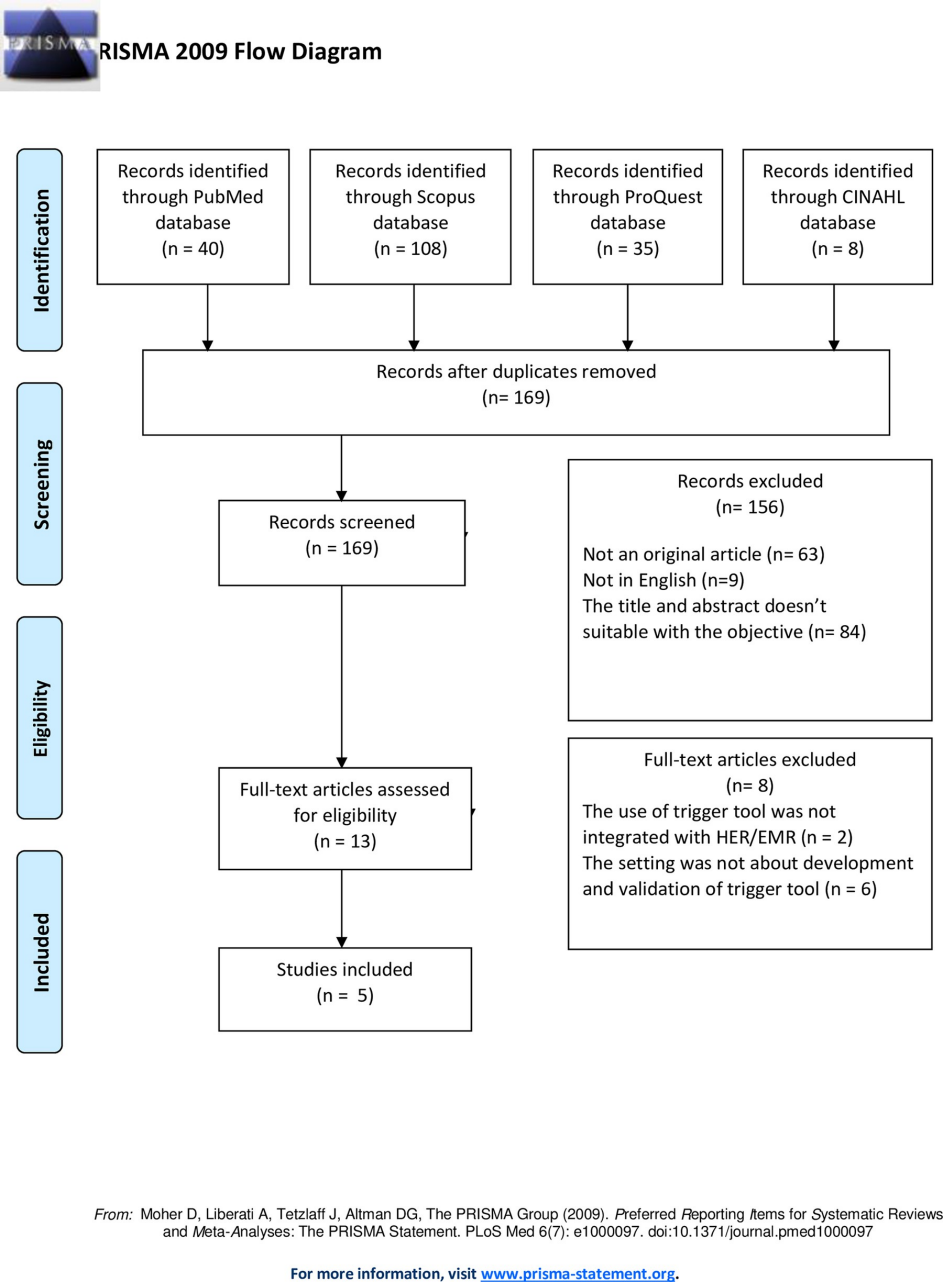


Studies that clearly describe the process development and validation of trigger tool in primary care or combination between primary and secondary care were considered eligible Furthermore, studies were included if the process development and validation of the trigger tool had an objectively defined outcome. However, due to the scarcity of research on this subject, a total of 196 articles were retrieved, with 6 duplications automatically removed using Mendeley Reference Manager. There were 190 records left after duplicates were removed. Ten full-text articles were determined to be eligible. Finally, five articles were chosen after passing the eligibility criteria. Fig 1 is a PRISMA flowchart depicting the entire study flow (S1 Fig PRISMA flowchart).

### Data extraction and synthesis

To address the review question systematically, relevant data were carefully extracted from the included studies using the methodological framework provided by the Joanna Briggs Institute (JBI) [13]. This extraction process included a wide range of information, such as the authors’ names, publication year, country of origin, and various study characteristics such as setting, study design, aim, significant findings, and outcomes, which are listed in Table 1 (S2 Table. Summary Table of Included Studies).

**Table 1:** Summary table of included studies.

Author, Year	Country and Setting	Aim	Study Design	Participants	Key Findings	Outcomes
Melle *et al*, 2018	Netherlands; Combined primary and secondary care	To pilot a review of medical records to identify transitional safety incidents (TSIs) for use in a large intervention study and assess its reliability and validity.	A cross-sectional retrospective medical record review study	301 patients that met the inclusion criteria	Development a. All items of in-hospital review medical record were reviewed and assessed on their fit for transitional patient safety. b. Reformulated questions to fit transitional patient safety. The unfit questions were deleted. c. The authors added items based on previously published pilot d. The resulting record review was then discussed in the research team, and after adjusting discussed with a wider expert team with GPs, hospital specialists and patients. e. Finally, the record review was tested two times by four reviewer on actual patient medical records Reliability To determine inter-rater reliability, a random sample of 10% of the medical records was reviewed by two reviewers independently. Validation a. Content validity: hospital specialists, GPs and an expert in medical record review studies were consulted to judge our TSI identification method on completeness and clarity, both individually during the review process and in a group discussion at the end of the review process. b. Concurrent validity: Due to the absence of a criterion measure in transitional patient safety, the study utilized three objectively identifiable Transitional Safety Incidents (TSIs) as a reference standard. These TSIs include the presence and timeliness of correspondence from hospital to general practitioner (GP), redundant diagnostic testing, and communication of in-hospital prescription changes to GP. Two layers review then was conducted by research assistant and research team.	The reviewers identified TSIs in 52 (17.3%) of all transitional medical records. Variation between reviewers was high (range: 3–28 per 50 medical records). Positive agreement for finding a TSI between reviewers was 0%, negative agreement 80% and the Cohen’s kappa −0.15. The reviewers identified 43 (22%) of 194 objectively identifiable TSIs.
Murphy *et al*, 2017	US, Combined hospital and clinic.	To develop, refine, and test trigger algorithms that identify patients with delayed follow-up evaluation of findings suspicious of colorectal cancer or hepatocellular cancer	A cross-sectional retrospective medical record review study of both delayed and non-delayed records to calculate trigger performance.	All patients seen in the inpatient and outpatient settings at all VA facilities nationwide	Development a. Designed the trigger to automatically exclude patients (exclusion criteria) and include patient with appropriate follow-up. b. The triggers must achieve a minimum of 50% positive predictive value (PPV) to be reviewed (for practical reason). c. The criteria were subsequently programmed into a combined computerized algorithm that was designed to extract structured data fields code. Validation a. Two reviewers performed manual chart reviews to determine whether the algorithm appropriately identified the intended information. b. Reviewers independently performed preliminary test record reviews and test the ability to correctly extract each criterion and evaluate output of the complete algorithm. c. Reviewers confirmed whether the data extracted by the trigger algorithm appropriately met the criteria.	CRC patients The study identified 1073 patients with delayed follow up from 245,158 CRC patients. The algorithm to identify patients with delayed follow-up in CRC cases has a positive predictive value (PPV) of 56.0% (95% CI, 51.0%–61.0%). The algorithm identified patients with delayed follow-up evaluation for CRC with 68.6% sensitivity (95% CI, 65.4%–71.6%) and 81.1% specificity (95% CI; 79.5%–82.6%) HCC patients From 333,828 patients, we identified 130 HCC patients with delayed follow up. The PPV of the algorithm was 82.3% (95% CI, 74.4%–88.2%). It identified patients with delayed follow-up evaluation for HCC with 89.1% sensitivity (95% CI, 81.8%–93.8%) and 96.5% specificity (95% CI, 94.8%–97.7%).
Knoll *et al*, 2022	US, Developing trigger tool in ambulatory care.	To design a trigger that would identify patients with an HbA1c over 10% who lacked appropriate follow-up HbA1c testing and compare this to the gold standard of chart review by clinicians.	A retrospective cohort study at a large, integrated health system using EHR data.	Patients age 18 years or older who met the eligible criteria	Development a. Defined and redefined denominator definitions representing all patient who were eligible for the care measure, and numerator definitions as those patients in the denominator who did not meet the care measure. b. Extracted a cohort of patients who met the initial denominator definition and performing detailed chart review. c. Clinician reviewed a random sample of cases that were triggers positive and to made recommendations on how to adjust the definition to reduce the false positive rate. d. The full multidisciplinary team met collaboratively to review these recommendations and the denominator and numerator were subsequently refined based on the discussion. e. This process of extraction, sampling of twenty charts, clinician review, multidisciplinary discussion, and denominator and numerator iteration was repeated until “saturation” was reached. Validation a. Reviewers were blinded as to whether the chart was trigger positive or trigger negative. These chart reviews were then used as the gold standard to determine the sensitivity, specificity, PPV, and NPV of the final algorithm as well as exact binomial confidence intervals for these estimates. b. Separated randomly patient charts that were reviewed for trigger validation into those that were trigger positive and those that were negative.	The final trigger had a high sensitivity and specificity and a PPV of 89% and a NPV of 100% for detection of delayed follow-up of HbA1c. The trigger detected 6228 patients had an HbA1c greater than 10%. Of these patients, 3131 (50.3%) were found to be trigger positive, while 3097 (49.7%) were trigger negative. Based on PPV and NPV of the trigger, we estimated that 2787 (95% CI 2313–3033) of trigger positive patients had no follow-up HbA1c result and 0 (95% CI 0–319) of trigger negative patients had a follow-up HbA1c result. The prevalence of delayed follow-up testing in the overall cohort was 45% (95% CI 33–57%). Compared to nonselective methods, use of the algorithm reduced the number of records required for review to identify a delay by more than 99%.
Suarez *et al*, 2020	US, combined primary and secondary care,	to validate the Elderly Risk Assessment (ERA) score as a predictor of hospitalization, mortality, and return visits in ED patients.	An observational cohort study	Patients age 60 years and older who presented to ED between January and December 2017.	Development a. The ERA tool was developed by identifying risk for adverse outcome that automatically calculated within the electronic health record (EHR). b. The ERA incorporates a weighted score of age, number of hospital days in the prior 2 years, marital status, medical diagnoses of congestive heart failure (CHF), myocardial infarction (MI), coronary artery disease (CAD), diabetes mellitus, cerebrovascular accident or stroke (CVA), chronic obstructive pulmonary disease (COPD), cancer, and dementia c. Identifying risk for adverse outcomes using ERA tools index will allow providers to deploy successful interventions to reduce return ED visits. Validation a. The validation process of the ERA tool was done by followed patients through health record review for 1 year from the index ED visit to determine whether there was a return visit and/or death. b. The validation process also was based on the ERA score that easily attainable and can be automatically calculated via the electronic medical record. c. The patients with ERA scores ≥ 16 were more likely to be admitted to the hospital, return to the ED within 30 days, and to die within one year. d. Connecting the concepts of validated screening tools for geriatric ED populations combined with proven interventions to decrease readmissions will improve the quality of life for our growing population of older patients.	The ERA score can be automatically calculated within the electronic medical record and can help identify older ED patients at higher risk for adverse outcomes, including death, hospitalization and return visits. Patients from 54% of visits were admitted to the hospital, 16% returned to the ED within 30 days, and 18% died within one year Higher ERA scores were associated with: hospital admission (score 10 [4–16] vs 5 [1–11], p < 0.0001), return visits (11 [5–17] vs 7 [2–13], p < 0.0001); and death within one year (14 [7–20] vs 6 [2–13], p < 0.0001). Patients with ERA score ≥ 16 were more likely to be admitted to the hospital OR 2.14 (2.02–2.28, p< 0,0001); return within 30 days OR 1.99 (1.85–2.14) and to die within a year OR 2,69 (2.54–2.85)
Walji, et al 2020	US, 4 large academic dental institutions.	To develop and determine how well the triggers performed in finding AEs and what characteristics dental AEs had in terms of type and severity.	A cross-sectional retrospective electronic dental patient charts.	All patients	Development 7 triggers were developed and implemented across 4 large academic dental institutions b. The triggers were implemented with structured query language and run against the institutional EHRs c. Some triggers also searched for keywords in the clinical notes d. Clinical team members identified keywords for inclusion criteria. e. An iterative process was used to run the triggers. A small sample of the resulting charts were reviewed and the triggers were further refined. f. Negation phrases in the trigger algorithm were included, to exclude some cases. g. After each trigger was executed, a list of patient charts meeting those criteria over a 1-y period was provided to the chart reviewers for further investigation. Validation a. We first used an automated trigger query to identify a set of patient charts that met the designated criteria. b. Two independent chart reviewers at each site then reviewed a random sample of triggered charts to determine the presence or absence of a dental AE. c. To estimate the projected number of triggered charts needed to review, we apply the sample size formula for proportions using initial values derived from pilot data d. Reviewers at each site convened to reach a consensus on every AE identified and used a REDCap form (Harris et al. 2009) to input their findings. e. To confirm our findings, we implemented a second level of review by an expert panel composed of calibrated investigators from each site. f. They first independently reviewed the AEs found during the individual site review; then, they met as a group and adjudicated every AE to make a final determination g. Once an AE was identified, reviewers categorized it by type (using a list of 12 items) and severity (using a 5-item scale)	a. In total, 3,658 patient charts were identified by the 7 triggers. b. A random sample of 1,885 charts were reviewed, and 305 charts (16.2%) contained an AE. c. Multiple AEs were found in the same chart, yielding a total of 324 AEs d. The individual site reviewers initially identified 490 AEs. The expert panel that further reviewed each of these events determined that 66% (n = 324) were actually AEs. e. The PPV ranged from a high of 0.23 for our 2 best-performing triggers (failed implants and postsurgical complications) to 0.09 for our lowest performing triggers (allergy/ toxicity and aspiration/ ingestion) f. The most common types of AEs found were pain (27.5%), hard tissue (14.8%), soft tissue (14.8%), and nerve injuries (13.3%)g. The severity of the AEs was most classified as temporary harm (89.2%). Permanent harm was present in 9.6% of the AEs, and 1.2% required transfer to an emergency room.

The extraction of study design was executed with precision to ascertain the rigor and relevance of the studies, adhering to the PO and PICO levels of evidence delineated by the Centre for Evidence-Based Medicine (CEBM) for a nuanced understanding of the research methodologies employed [15]. In order to map the included research, we emphasized on the complexities of process development and validation in the context of trigger tool implementation. Furthermore, key findings pertaining to the aforementioned processes were systematically extracted to summarize the essence of the studies. This entailed a careful examination of the complexities involved in the creation and validation of trigger tools, shedding light on the differences and challenges encountered. Moreover, the outcomes measured in each of the five selected studies were extracted, allowing for a more comprehensive synthesis of the findings and increasing the overall depth and clarity of the review.

## Results

The final review included five articles. The studies were carried out in various regions, including the United States [16–19] and the Netherlands [20]. We examined the included studies to determine the main findings about the process development and validation of the trigger tool. S1 File contains a detailed extraction of the study (S1 File Detailed extraction of the study).

### Study characteristics

The majority of the studies were cross-sectional [16, 19, 20] with the other being a cohort retrospective study [17–19]. Setting of the studies were in primary care, [17–19] or in a combination of primary and secondary care [16, 20]. We include studies in combined primary-secondary care to gain more findings due to a lack of recent studies on process development and validation with focused settings in primary care. The majority of the studies had similar contexts, but the objectives varied. There are two studies with the goal of determining the process development of a trigger tool [16, 17]. Other studies’ goals include identifying process development and validation [18], process development and implementation [19], and asses the validity and reliability process of the trigger tool [20].

### Process development of trigger tool

The findings indicated there were some studies about the process development of trigger tool both in primary care and secondary care settings. The first step in developing trigger tools in primary care was to clearly define the risk, red flag criteria, or trigger [16, 19]. In order to make triggers more practical, clinicians must also define exclusion criteria and appropriate follow-up criteria [16]. The first step toward trigger development was consistent measurements of the trigger tool criteria, so that the number and type of AEs detected would be more meaningful [19]. These criteria were then programmed into computerized algorithm designed to extract arranged data fields [18].

To develop the trigger tool, the research have to repeatedly define and redefine the denominator and numerator definitions that representing all patients [17]. The denominator means patients who were eligible for the care measures and the other who did not eligible were define as the numerator [17]. Using the extracted a cohort data of patients who met the initial denominator, a clinician then can reviewed a random sample of cases that were trigger positive. Identified as trigger positives was when the patient electronic medical records met both the initial numerator and denominator criteria [16, 17].

The trigger tool was created using a computerized algorithm or an automated rule based approach based on information from the patient’s electronic medical record [18]. The recognized drug-AE pairs were extracted if they met the pre-defined criteria and trigger phrase rules [16, 17]. The criteria were then programmed into a computerized algorithm designed to extract structured data fields code [16]. It was also necessary to conduct an error analysis on some charts that were falsely triggered when developing trigger tools [16, 19]. As it can be used to boost the predictive value score [18]. To be considered in detected AE, the triggers must have a positive predictive value (PPV) of at least 50 percent [16].

### Validation of trigger tool

According to the analysis results, after the patient electronic medical records (EMRs) screening process, it must be reviewed or re-validated before identified as positive for AEs. The content and concurrent validity can be focused on during the review or validity process [20]. Content validity was defined as identifying patients’ EMR based on the instrument’s intended measurement. The reviewers evaluate the completeness and clarity of the review process both individually and in a group discussion [20]. Concurrent validity is defined as identifying scores based on criterion measure scores administered at the same time [18, 20]. The score is calculated automatically within the EMR and is used in daily practice in primary care, with higher scores being more likely to be admitted as an adverse event [18, 20].

Some studies demonstrate another method of reviewing or validating the EMR by randomly or blindly selecting whether the chart was trigger positive or trigger negative [17, 19]. Two experts then performed an in-depth chart review to determine the final algorithm’s sensitivity, specificity, positive predictive value (PPV), and negative predictive value (NPV) [19]. Following that, the reviewers randomly separated the patient charts that had been reviewed or validated for trigger positive and trigger negative. The PPV was calculated by confirming the number of ADE with exact binomial confidence intervals [17, 19].

Experts performed a manual chart review to determine whether the algorithm correctly identified the intended information [16, 19]. Reviewers independently performed a preliminary test record review, assessed the ability to correctly extract each criterion, and evaluated the output of the entire trigger tool algorithm [19]. Each summary was evaluated by some expert reviewers to ensure the reliability of AE that were automatically recognized with the trigger tool. Furthermore, expert reviewers from both primary care and research confirmed that the data extracted by the trigger algorithm met the criteria [16, 18].

## Discussion

Despite its importance, studies related to the development and validation of trigger tools in primary care are scarce. Therefore, we also examined combined settings to gather additional information. This scoping review discovered some similarities in the development and validation of trigger tools in both primary and combined primary and secondary care settings. In regards the triggers development, the primary process was started by clearly define the triggers or red flag criteria that were eligible for care measures and those that were not, based on the patient medical record [16, 19]. Triggers are flags or prompts discovered during a review of the medical record that prompt further investigation to determine the presence or absence of an adverse event [21]. Each trigger had its own adverse event analysis flowchart that described the criteria required to confirm or deny the occurrence of AEs when the trigger was detected [22]. To make the trigger more practical, the exclusion and appropriate follow-up criteria need to be clearly defined [16, 17]. The criteria were then programmed into a computerized algorithm, and drug-AE pairs that met the pre-defined criteria and trigger phrase rules were detected [16–18]. The AEs were more likely to be detected with high scores calculated automatically from the patients’ EMR, resulting in poor outcomes or even death [23, 24].

We also discovered some new ways to define trigger criteria from studies conducted in primary care settings by analyzing extracted cohort data from patients who met the initial denominator [17]. Clinicians must define and redefine the denominator and numerator definitions that represent all patients on a regular basis [17]. The denominator refers to patients who were eligible for the care measures, while the numerator refers to those who were not [17]. Another study conducted at the Children’s National Hospital used the same method to define the triggers criteria where clinicians devised a numerator and denominator measure to quantify trigger criteria [25]. The numerator was the percentage of cases that represented true AEs or near-miss events, and the denominator was the total number of triggers activated [25]. In contrast, a Chinese study used a literature review and Delphi method to develop ADE trigger tools for Chinese geriatric inpatients [26, 27]. Triggers were developed based on the literature review and clinical logic, then adapted to local healthcare settings. The next step was a Delphi panel, which was a structured and reliable method for gathering the opinions of an expert group in order to make a decision. Those triggers were first revised and presented to the two-round Delphi panel for revision and rating as part of the validation process [26, 27].

The validation approach was similar across most studies, involving manual chart reviews conducted by experts, including both physicians and non-physicians, with a specific emphasis on content and concurrent validity. In prior research conducted in combined hospitals and clinics in the mid-western US, red flags were defined, and algorithms were developed to identify them from patient data, thereby creating electronic "triggers" for diagnostic delays [28]. Additionally, the validation of trigger tools involved experts applying each criterion to the database and conducting a manual review of trigger-positive patient records to ensure accurate identification of the relevant information [29]. Following validation, all criteria were compiled into a stepwise algorithm that identified red flags, excluded clinical exclusion criteria, and determined appropriate follow-up criteria. These electronic trigger algorithms have demonstrated success in detecting delays in the follow up of red flags indicative of colorectal, hepatocellular, bladder, lung, and prostate cancer [30]. This underscores the effectiveness of the validation approach in ensuring the accuracy and reliability of trigger tools, particularly in the context of detecting diagnostic delays for various types of cancer.

The majority of previous attempts at validating trigger tools focused on positive predictive value [31]. Trigger tools with high positive predictive values are critical in reducing false positive rates, as long as their sensitivity is kept to a reasonable level [32]. This metric is useful for estimating the yield within the flagged records as well as the excess workload caused by false-positive flags [33]. The more sensitive the trigger tool, the more likely it will be developed using clinical decision rule methodology on the process of trigger tool development [34].

The process of developing and validating the trigger tool was also comparable to research done in hospital settings in Sweden and Singapore [12, 35, 36]. In Sweden, a pediatric trigger tool was developed with a broad literature review and expert opinion as validation using a modified Delphi process. Then, in Singapore, the process of developing mental health trigger tools was based on existing literature to develop the trigger list [36]. Furthermore, there were two stages of review during the validation process. For each trigger detected in the patient’s medical record, all records were reviewed by the respective registered nurse or pharmacist (non-physicians) [12, 35, 36]. The physicians then conducted a final independent review to validate the records that had been identified as having ADEs. Another study in Canada used a different validation process in hospital settings [32]. It indicates that the trigger tools were validated by comparing adverse events identified on the tools to events identified by clinical care providers at the point of care (retrospective methods). The significance of validating retrospective adverse event case finding methods against a robust prospective standard was to allow for refinement prior to widespread implementation [32].

The development and validation of trigger tools resulted in a promising and efficient safety review in emergency medicine in order to detect all harm from patient medical records [37]. Trigger tools have been developed in various clinical settings and countries, with excellent results as surveillance tools for detecting errors [38, 39]. Trigger tools are intended for AE surveillance in order to detect all causes of harm, both preventable and unpreventable [37, 38]. As a result, this greatly assists clinicians in preventing treatment errors that can result in adverse events [40]. The development of trigger tools could detect a broader range of events, allowing for the establishment of a baseline for assessing and allocating resources to improve quality care and patient safety [38]. Triggers that are detected will prompt a detailed review to look for evidence during the validation process, resulting in significant reductions in false errors [37, 40].

Recognizing the study’s limitations is critical, and one notable limitation is the scarcity of research conducted in primary care settings. This limitation stems from the use of a limited set of keywords as well as the limitation of databases to English-language papers. It is critical to understand that this focus may inadvertently exclude valuable insights from non-English sources and alternative terminologies. Despite these limitations, the study makes an important contribution by including updated information on the development and validation processes in both primary care and combined primary-secondary care settings. The inclusion of a time frame (within the last 5 years) ensures the data’s relevance and currency. Furthermore, the study adheres to the Centre for Evidence-Based Medicine’s (CEBM) rigorous standards, incorporating levels of evidence based on the PO (Patient/Population, Outcome) and PICO (Patient/Population, Intervention, Comparison, Outcome) frameworks. This methodological approach improves the analysis’s robustness and reliability.

## Conclusion

This study outlined a thorough process for developing trigger tools, emphasizing the critical step of clearly defining triggers or red flag criteria through an extensive literature review. A critical aspect of ensuring accuracy is the validation process, which includes a dual evaluation by non-physician professionals followed by a final validation by physicians. The emphasis on identifying triggers with positive predictive value is consistent with the use of clinical decision rule methodology, which improves the precision and reliability of trigger tools in detecting adverse events. This study emphasized the similarities between the processes of developing and validating trigger tools in primary care and secondary care or hospital settings. The similarity of these processes suggests that primary care can benefit from the methodologies used in hospital settings. Primary care practitioners can improve their understanding and application of trigger tools by drawing parallels, contributing to improved patient safety and proactive adverse event detection.

## Supporting information

S1 Table Search terms. (DOCX)

S2 Table Summary table of included studies. (DOCX)

S1 Fig PRISMA flowchart. (TIF)

S1 File Detailed extraction of the study. (DOCX)

S2 File Preferred Reporting Items for Systematic reviews and Meta-Analyses extension for Scoping Reviews (PRISMA-ScR) checklist. (DOCX)
