# Supplementary material for: Development and validation of trigger tools in primary care: A scoping review
Source: PLoS One. 2025 Jan 2;20(1):e0308906. doi: 10.1371/journal.pone.0308906 (PMC11694991; doi:10.1371/journal.pone.0308906)
Supplement: S1 Table — (DOCX) [file pone.0308906.s001.docx]

Supplementary 1

**Table 1.** Search Terms.

| **Population** | **Intervention** | **Concept** | **Outcome** |
| --- | --- | --- | --- |
| Primary care | Development | Electronic medical record | Global trigger tool |
| Public health center | Development validation | Electronic health  record | Automated trigger tool |
| Ambulatory care |  | |  |
| Outpatient clinic |  |  |  |
