# Supplementary material for: Development and validation of trigger tools in primary care: A scoping review
Source: PLoS One. 2025 Jan 2;20(1):e0308906. doi: 10.1371/journal.pone.0308906 (PMC11694991; doi:10.1371/journal.pone.0308906)
Supplement: S2 Table — (DOCX) [file pone.0308906.s002.docx]

Table 2. Summary Table of Included Studies

| **Author, Year** | **Country and Setting** | **Aim** | **Study Design** | **Participants** | **Key Findings** | **Outcomes** |
| --- | --- | --- | --- | --- | --- | --- |
| Melle *et al,* 2018 | Netherlands; Combined primary and secondary care | To pilot a review of medical records to identify transitional safety incidents (TSIs) for use in a large intervention study and assess its reliability and validity. | A cross-sectional retrospective medical record review study | 301 patients that met the inclusion criteria | Development   1. All items of in-hospital review medical record were reviewed and assessed on their fit for transitional patient safety. 2. Reformulated questions to fit transitional patient safety. The unfit questions were deleted. 3. The authors added items based on previously published pilot 4. The resulting record review was then discussed in the research team, and after adjusting discussed with a wider expert team with GPs, hospital specialists and patients. 5. Finally, the record review was tested two times by four reviewer on actual patient medical records   Reliability  To determine inter-rater reliability, a random sample of 10% of the medical records was reviewed by two reviewers independently.  Validation   1. Content validity: hospital specialists, GPs and an expert in medical record review studies were consulted to judge our TSI identification method on completeness and clarity, both individually during the review process and in a group discussion at the end of the review process. 2. Concurrent validity: Due to the absence of a criterion measure in transitional patient safety, the study utilized three objectively identifiable Transitional Safety Incidents (TSIs) as a reference standard. These TSIs include the presence and timeliness of correspondence from hospital to general practitioner (GP), redundant diagnostic testing, and communication of in-hospital prescription changes to GP. Two layers review then was conducted by research assistant and research team. | 1. The reviewers identified TSIs in 52 (17.3%) of all transitional medical records. 2. Variation between reviewers was high (range: 3–28 per 50 medical records). 3. Positive agreement for finding a TSI between reviewers was 0%, negative agreement 80% and the Cohen’s kappa −0.15. 4. The reviewers identified 43 (22%) of 194 objectively identifiable TSIs. |
| Murphy *et al,* 2017 | US, Combined hospital and clinic. | To develop, refine, and test trigger algorithms that identify patients with delayed follow-up evaluation of findings suspicious of colorectal cancer or hepatocellular cancer | A cross-sectional retrospective medical record review study of both delayed and non-delayed records to calculate trigger performance. | All  patients seen in the inpatient and outpatient settings at all VA facilities nationwide | Development   1. Designed the trigger to automatically exclude patients (exclusion criteria) and include patient with appropriate follow-up. 2. The triggers must achieve a minimum of 50% positive predictive value (PPV) to be reviewed (for practical reason). 3. The criteria were subsequently programmed into a combined computerized algorithm that was designed to extract structured data fields code.   Validation   1. Two reviewers performed manual chart reviews to determine whether the algorithm appropriately identified the intended information. 2. Reviewers independently performed preliminary test record reviews and test the ability to correctly extract each criterion and evaluate output of the complete algorithm. 3. Reviewers confirmed whether the data extracted by the trigger algorithm appropriately met the criteria. | CRC patients   1. The study identified 1073 patients with delayed follow up from 245,158 CRC patients. 2. The algorithm to identify patients with delayed follow-up in CRC cases has a positive predictive value (PPV) of 56.0% (95% CI, 51.0%–61.0%). 3. The algorithm identified patients with delayed follow-up evaluation for CRC with 68.6% sensitivity (95% CI, 65.4%–71.6%) and 81.1% specificity (95% CI; 79.5%–82.6%)   HCC patients   1. From 333,828 patients, we identified 130 HCC patients with delayed follow up. 2. The PPV of the algorithm was 82.3% (95% CI, 74.4%–88.2%). 3. It identified patients with delayed follow-up evaluation for HCC with 89.1% sensitivity (95% CI, 81.8%–93.8%) and 96.5% specificity (95% CI, 94.8%–97.7%). |
| Knoll *et al,* 2022 | US, Developing trigger tool in ambulatory care. | To design a trigger that would identify patients with an HbA1c over 10% who lacked appropriate follow-up HbA1c testing and compare this to the gold standard of chart review by clinicians. | A retrospective cohort study at a large, integrated health system using EHR data. | Patients age 18 years or older who met the eligible criteria | Development   1. Defined and redefined denominator definitions representing all patient who were eligible for the care measure, and numerator definitions as those patients in the denominator who did not meet the care measure. 2. Extracted a cohort of patients who met the initial denominator definition and performing detailed chart review. 3. Clinician reviewed a random sample of cases that were triggers positive and to made recommendations on how to adjust the definition to reduce the false positive rate. 4. The full multidisciplinary team met collaboratively to review these recommendations and the denominator and numerator were subsequently refined based on the discussion. 5. This process of extraction, sampling of twenty charts, clinician review, multidisciplinary discussion, and denominator and numerator iteration was repeated until “saturation” was reached.   Validation   1. Reviewers were blinded as to whether the chart was trigger positive or trigger negative. These chart reviews were then used as the gold standard to determine the sensitivity, specificity, PPV, and NPV of the final algorithm as well as exact binomial confidence intervals for these estimates. 2. Separated randomly patient charts that were reviewed for trigger validation into those that were trigger positive and those that were negative. | 1. The final trigger had a high sensitivity and specificity and a PPV of 89% and a NPV of 100% for detection of delayed follow-up of HbA1c. 2. The trigger detected 6228 patients had an HbA1c greater than 10%. Of these patients, 3131 (50.3%) were found to be trigger positive, while 3097 (49.7%) were trigger negative. 3. Based on PPV and NPV of the trigger, we estimated that 2787 (95% CI 2313–3033) of trigger positive patients had no follow-up HbA1c result and 0 (95% CI 0–319) of trigger negative patients had a follow-up HbA1c result. 4. The prevalence of delayed follow-up testing in the overall cohort was 45% (95% CI 33–57%). 5. Compared to nonselective methods, use of the algorithm reduced the number of records required for review to identify a delay by more than 99%. |
| Suarez *et al,* 2020 | US, combined primary and secondary care, | to validate the Elderly Risk Assessment (ERA) score as a predictor of hospitalization, mortality, and return visits in ED patients. | An observational cohort study | Patients age 60 years and older who presented to ED between January and December 2017. | Development   1. The ERA tool was developed by identifying risk for adverse outcome that automatically calculated within the electronic health record (EHR). 2. The ERA incorporates a weighted score of age, number of hospital days in the prior 2 years, marital status, medical diagnoses of congestive heart failure (CHF), myocardial infarction (MI), coronary artery disease (CAD), diabetes mellitus, cerebrovascular accident or stroke (CVA), chronic obstructive pulmonary disease (COPD), cancer, and dementia 3. Identifying risk for adverse outcomes using ERA tools index will allow providers to deploy successful interventions to reduce return ED visits.   Validation   1. The validation process of the ERA tool was done by followed patients through health record review for 1 year from the index ED visit to determine whether there was a return visit and/or death. 2. The validation process also was based on the ERA score that easily attainable and can be automatically calculated via the electronic medical record. 3. The patients with ERA scores ≥ 16 were more likely to be admitted to the hospital, return to the ED within 30 days, and to die within one year. 4. Connecting the concepts of validated screening tools for geriatric ED populations combined with proven interventions to decrease readmissions will improve the quality of life for our growing population of older patients. | 1. The ERA score can be automatically calculated within the electronic medical record and can help identify older ED patients at higher risk for adverse outcomes, including death, hospitalization and return visits. 2. Patients from 54% of visits were admitted to the hospital, 16% returned to the ED within 30 days, and 18% died within one year 3. Higher ERA scores were associated with: hospital admission (score 10 [4-16] vs 5 [1-11], p < 0.0001), return visits (11 [5-17] vs 7 [2-13], p < 0.0001); and death within one year (14 [7-20] vs 6 [2-13], p < 0.0001). 4. Patients with ERA score ≥ 16 were more likely to be admitted to the hospital OR 2.14 (2.02-2.28, p< 0,0001); return within 30 days OR 1.99 (1.85-2.14) and to die within a year OR 2,69 (2.54-2.85) |
| Walji, et al 2020 | US, 4 large academic dental institutions. | To develop and determine how well the triggers performed in finding AEs and what characteristics dental AEs had in terms of type and severity. | A cross-sectional retrospective electronic dental patient charts. | All patients | Development   1. 7 triggers were developed and implemented across 4 large academic dental institutions 2. The triggers were implemented with structured query language and run against the institutional EHRs 3. Some triggers also searched for keywords in the clinical notes 4. Clinical team members identified keywords for inclusion criteria. 5. An iterative process was used to run the triggers. A small sample of the resulting charts were reviewed and the triggers were further refined. 6. Negation phrases in the trigger algorithm were included, to exclude some cases. 7. After each trigger was executed, a list of patient charts meeting those criteria over a 1-y period was provided to the chart reviewers for further investigation.   Validation   1. We first used an automated trigger query to identify a set of patient charts that met the designated criteria. 2. Two independent chart reviewers at each site then reviewed a random sample of triggered charts to determine the presence or absence of a dental AE. 3. To estimate the projected number of triggered charts needed to review, we apply the sample size formula for proportions using initial values derived from pilot data 4. Reviewers at each site convened to reach a consensus on every AE identified and used a REDCap form (Harris et al. 2009) to input their findings. 5. To confirm our findings, we implemented a second level of review by an expert panel composed of calibrated investigators from each site. 6. They first independently reviewed the AEs found during the individual site review; then, they met as a group and adjudicated every AE to make a final determination 7. Once an AE was identified, reviewers categorized it by type (using a list of 12 items) and severity (using a 5-item scale; see Appendix 3). | 1. In total, 3,658 patient charts were identified by the 7 triggers. 2. A random sample of 1,885 charts were reviewed, and 305 charts (16.2%) contained an AE. 3. Multiple AEs were found in the same chart, yielding a total of 324 AEs 4. The individual site reviewers initially identified 490 AEs. The expert panel that further reviewed each of these events determined that 66% (n = 324) were actually AEs. 5. The PPV ranged from a high of 0.23 for our 2 best-performing triggers (failed implants and postsurgical complications) to 0.09 for our lowest performing triggers (allergy/ toxicity and aspiration/ ingestion) 6. The most common types of AEs found were pain (27.5%), hard tissue (14.8%), soft tissue (14.8%), and nerve injuries (13.3%) 7. The severity of the AEs was most classified as temporary harm (89.2%). Permanent harm was present in 9.6% of the AEs, and 1.2% required transfer to an emergency room. |
