# Supplementary figures and images for: Development and validation of trigger tools in primary care: A scoping review
Source: PLoS One. 2025 Jan 2;20(1):e0308906. doi: 10.1371/journal.pone.0308906 (PMC11694991; doi:10.1371/journal.pone.0308906)

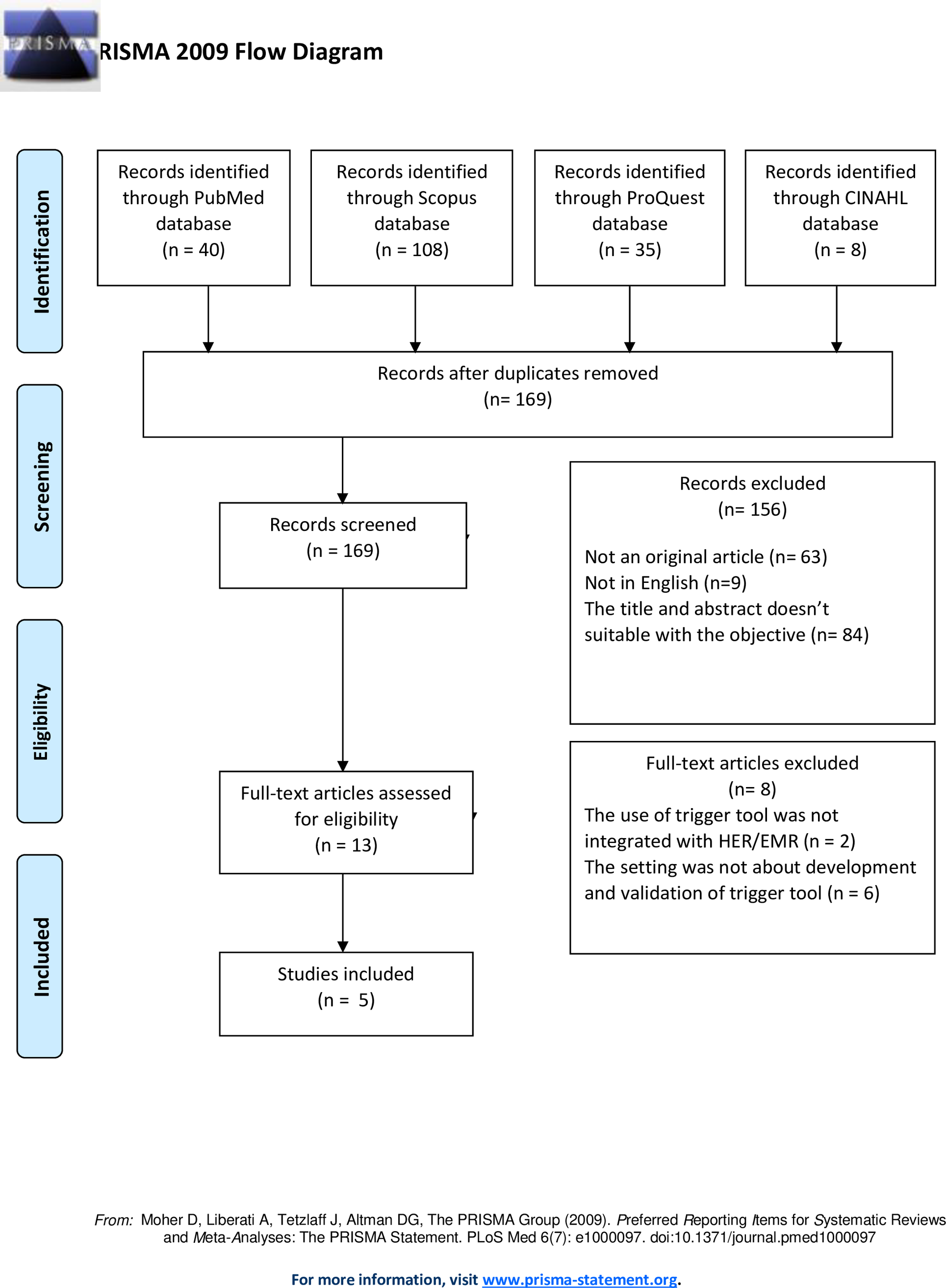

Supplement: S1 Fig — (TIF) [file pone.0308906.s003.tif]
