# Supplementary material for: Development and validation of trigger tools in primary care: A scoping review
Source: PLoS One. 2025 Jan 2;20(1):e0308906. doi: 10.1371/journal.pone.0308906 (PMC11694991; doi:10.1371/journal.pone.0308906)
Supplement: S1 File — (DOCX) [file pone.0308906.s004.docx]

Supplementary file 4

Detailed Extraction of the study

The final queries applied for each database could be seen below:

1. CINAHL

| # | Query | Limiters/Expanders Last Run Via | Results |
| --- | --- | --- | --- |
| S5 | (S1 AND S2 AND S3 AND S4) | Expanders - Apply Interface - EBSCOhost | 8 |
|  |  | equivalent subjects Research Databases |  |
|  |  | Search modes - Search Screen - Advanced |  |
|  |  | SmartText Searching Search |  |
|  |  | Database - CINAHL |  |
|  |  |  |  |
| S4 | global trigger tool OR | Limiters - Publication Interface - EBSCOhost | 3 |
|  | automated trigger tool | Date: 19240101- Research Databases |  |
|  |  | 20231031 Search Screen - Advanced |  |
|  |  | Expanders - Apply Search |  |
|  |  | equivalent subjects Database - CINAHL |  |
|  |  | Search modes - Find all |  |
|  |  | my search terms |  |
| S3 | electronic medical record | Limiters - Publication Interface - EBSCOhost | 1035 |
|  | OR electronic health | Date: 19240101- Research Databases |  |
|  | record | 20231031 Search Screen - Advanced |  |
|  |  | Expanders - Apply Search |  |
|  |  | equivalent subjects Database - CINAHL |  |
|  |  | Search modes - Find all |  |
|  |  | my search terms |  |
| S2 | development OR | Limiters - Publication Interface - EBSCOhost | 49552 |
|  | development validation | Date: 19240101- Research Databases |  |
|  |  | 20231031 Search Screen - Advanced |  |
|  |  | Expanders - Apply Search |  |
|  |  | equivalent subjects Database - CINAHL |  |
|  |  | Search modes - Find all |  |
|  |  | my search terms |  |
| S1 | primary care OR public | Limiters - Publication Interface - EBSCOhost | 18823 |
|  | health center OR | Date: 19240101- Research Databases |  |
|  | ambulatory care OR | 20231031 Search Screen - Advanced |  |
|  | outpatient clinic | Expanders - Apply Search |  |
|  |  | equivalent subjects Database - CINAHL |  |
|  |  | Search modes - Find all |  |
|  |  | my search terms |  |

1. Pubmed

| Search number | Query | Sort By | Filters | Search Details | Results | Time |
| --- | --- | --- | --- | --- | --- | --- |
| 5 | ((((((primary care) OR (public health center)) OR (ambulatory care)) OR (outpatient clinic)) AND ((development) OR (development validation))) AND ((electronic medical record) OR (electronic health record))) AND ((global trigger tool) OR (automated trigger tool)) | Most Recent | English | ("primary health care"[MeSH Terms] OR ("primary"[All Fields] AND "health"[All Fields] AND "care"[All Fields]) OR "primary health care"[All Fields] OR ("primary"[All Fields] AND "care"[All Fields]) OR "primary care"[All Fields] OR (("public health"[MeSH Terms] OR ("public"[All Fields] AND "health"[All Fields]) OR "public health"[All Fields]) AND ("center"[All Fields] OR "center s"[All Fields] OR "centers"[All Fields] OR "centre"[All Fields] OR "centre s"[All Fields] OR "centres"[All Fields])) OR ("ambulatory care"[MeSH Terms] OR ("ambulatory"[All Fields] AND "care"[All Fields]) OR "ambulatory care"[All Fields]) OR ("ambulatory care facilities"[MeSH Terms] OR ("ambulatory"[All Fields] AND "care"[All Fields] AND "facilities"[All Fields]) OR "ambulatory care facilities"[All Fields] OR ("outpatient"[All Fields] AND "clinic"[All Fields]) OR "outpatient clinic"[All Fields])) AND ("develop"[All Fields] OR "develope"[All Fields] OR "developed"[All Fields] OR "developer"[All Fields] OR "developer s"[All Fields] OR "developers"[All Fields] OR "developing"[All Fields] OR "developments"[All Fields] OR "develops"[All Fields] OR "growth and development"[MeSH Subheading] OR ("growth"[All Fields] AND "development"[All Fields]) OR "growth and development"[All Fields] OR "development"[All Fields] OR (("develop"[All Fields] OR "develope"[All Fields] OR "developed"[All Fields] OR "developer"[All Fields] OR "developer s"[All Fields] OR "developers"[All Fields] OR "developing"[All Fields] OR "developments"[All Fields] OR "develops"[All Fields] OR "growth and development"[MeSH Subheading] OR ("growth"[All Fields] AND "development"[All Fields]) OR "growth and development"[All Fields] OR "development"[All Fields]) AND ("valid"[All Fields] OR "validate"[All Fields] OR "validated"[All Fields] OR "validates"[All Fields] OR "validating"[All Fields] OR "validation"[All Fields] OR "validational"[All Fields] OR "validations"[All Fields] OR "validator"[All Fields] OR "validators"[All Fields] OR "validities"[All Fields] OR "validity"[All Fields]))) AND ("electronic health records"[MeSH Terms] OR ("electronic"[All Fields] AND "health"[All Fields] AND "records"[All Fields]) OR "electronic health records"[All Fields] OR ("electronic"[All Fields] AND "medical"[All Fields] AND "record"[All Fields]) OR "electronic medical record"[All Fields] OR ("electronic health records"[MeSH Terms] OR ("electronic"[All Fields] AND "health"[All Fields] AND "records"[All Fields]) OR "electronic health records"[All Fields] OR ("electronic"[All Fields] AND "health"[All Fields] AND "record"[All Fields]) OR "electronic health record"[All Fields])) AND ((("global"[All Fields] OR "globalism"[All Fields] OR "globalize"[All Fields] OR "globalized"[All Fields] OR "globalizes"[All Fields] OR "globalizing"[All Fields] OR "globally"[All Fields] OR "globals"[All Fields] OR "internationality"[MeSH Terms] OR "internationality"[All Fields] OR "globalization"[All Fields]) AND ("precipitating factors"[MeSH Terms] OR ("precipitating"[All Fields] AND "factors"[All Fields]) OR "precipitating factors"[All Fields] OR "trigger"[All Fields] OR "triggers"[All Fields] OR "triggerable"[All Fields] OR "triggered"[All Fields] OR "triggering"[All Fields] OR "triggerings"[All Fields]) AND "tool"[All Fields]) OR (("automate"[All Fields] OR "automated"[All Fields] OR "automates"[All Fields] OR "automating"[All Fields] OR "automation"[MeSH Terms] OR "automation"[All Fields] OR "automations"[All Fields] OR "automation s"[All Fields]) AND ("precipitating factors"[MeSH Terms] OR ("precipitating"[All Fields] AND "factors"[All Fields]) OR "precipitating factors"[All Fields] OR "trigger"[All Fields] OR "triggers"[All Fields] OR "triggerable"[All Fields] OR "triggered"[All Fields] OR "triggering"[All Fields] OR "triggerings"[All Fields]) AND "tool"[All Fields])) | 40 | 0:32:28 |
| 4 | (global trigger tool) OR (automated trigger tool) | Most Recent |  | (("global"[All Fields] OR "globalism"[All Fields] OR "globalize"[All Fields] OR "globalized"[All Fields] OR "globalizes"[All Fields] OR "globalizing"[All Fields] OR "globally"[All Fields] OR "globals"[All Fields] OR "internationality"[MeSH Terms] OR "internationality"[All Fields] OR "globalization"[All Fields]) AND ("precipitating factors"[MeSH Terms] OR ("precipitating"[All Fields] AND "factors"[All Fields]) OR "precipitating factors"[All Fields] OR "trigger"[All Fields] OR "triggers"[All Fields] OR "triggerable"[All Fields] OR "triggered"[All Fields] OR "triggering"[All Fields] OR "triggerings"[All Fields]) AND "tool"[All Fields]) OR (("automate"[All Fields] OR "automated"[All Fields] OR "automates"[All Fields] OR "automating"[All Fields] OR "automation"[MeSH Terms] OR "automation"[All Fields] OR "automations"[All Fields] OR "automation s"[All Fields]) AND ("precipitating factors"[MeSH Terms] OR ("precipitating"[All Fields] AND "factors"[All Fields]) OR "precipitating factors"[All Fields] OR "trigger"[All Fields] OR "triggers"[All Fields] OR "triggerable"[All Fields] OR "triggered"[All Fields] OR "triggering"[All Fields] OR "triggerings"[All Fields]) AND "tool"[All Fields]) | 742 | 0:32:05 |
| 3 | (electronic medical record) OR (electronic health record) | Most Recent |  | "electronic health records"[MeSH Terms] OR ("electronic"[All Fields] AND "health"[All Fields] AND "records"[All Fields]) OR "electronic health records"[All Fields] OR ("electronic"[All Fields] AND "medical"[All Fields] AND "record"[All Fields]) OR "electronic medical record"[All Fields] OR ("electronic health records"[MeSH Terms] OR ("electronic"[All Fields] AND "health"[All Fields] AND "records"[All Fields]) OR "electronic health records"[All Fields] OR ("electronic"[All Fields] AND "health"[All Fields] AND "record"[All Fields]) OR "electronic health record"[All Fields]) | 93,372 | 0:31:13 |
| 2 | (development) OR (development validation) | Most Recent |  | "develop"[All Fields] OR "develope"[All Fields] OR "developed"[All Fields] OR "developer"[All Fields] OR "developer s"[All Fields] OR "developers"[All Fields] OR "developing"[All Fields] OR "developments"[All Fields] OR "develops"[All Fields] OR "growth and development"[MeSH Subheading] OR ("growth"[All Fields] AND "development"[All Fields]) OR "growth and development"[All Fields] OR "development"[All Fields] OR (("develop"[All Fields] OR "develope"[All Fields] OR "developed"[All Fields] OR "developer"[All Fields] OR "developer s"[All Fields] OR "developers"[All Fields] OR "developing"[All Fields] OR "developments"[All Fields] OR "develops"[All Fields] OR "growth and development"[MeSH Subheading] OR ("growth"[All Fields] AND "development"[All Fields]) OR "growth and development"[All Fields] OR "development"[All Fields]) AND ("valid"[All Fields] OR "validate"[All Fields] OR "validated"[All Fields] OR "validates"[All Fields] OR "validating"[All Fields] OR "validation"[All Fields] OR "validational"[All Fields] OR "validations"[All Fields] OR "validator"[All Fields] OR "validators"[All Fields] OR "validities"[All Fields] OR "validity"[All Fields])) | 6,405,568 | 0:30:19 |
| 1 | (((primary care) OR (public health center)) OR (ambulatory care)) OR (outpatient clinic) | Most Recent |  | "primary health care"[MeSH Terms] OR ("primary"[All Fields] AND "health"[All Fields] AND "care"[All Fields]) OR "primary health care"[All Fields] OR ("primary"[All Fields] AND "care"[All Fields]) OR "primary care"[All Fields] OR (("public health"[MeSH Terms] OR ("public"[All Fields] AND "health"[All Fields]) OR "public health"[All Fields]) AND ("center"[All Fields] OR "center s"[All Fields] OR "centers"[All Fields] OR "centre"[All Fields] OR "centre s"[All Fields] OR "centres"[All Fields])) OR ("ambulatory care"[MeSH Terms] OR ("ambulatory"[All Fields] AND "care"[All Fields]) OR "ambulatory care"[All Fields]) OR ("ambulatory care facilities"[MeSH Terms] OR ("ambulatory"[All Fields] AND "care"[All Fields] AND "facilities"[All Fields]) OR "ambulatory care facilities"[All Fields] OR ("outpatient"[All Fields] AND "clinic"[All Fields]) OR "outpatient clinic"[All Fields]) | 3,366,257 | 0:26:55 |

1. Scopus

| Search Strategy |  |  |  |  |  |  |  |  |  |  |
| --- | --- | --- | --- | --- | --- | --- | --- | --- | --- | --- |
|  |  |  |  |  |  |  |  |  |  |  |
| Set#: Search 1 |  |  |  |  |  |  |  |  |  |  |
| Searched for: "primary care" OR "public health center" OR "ambulatory care" OR "outpatient clinic" |  |  |  |  |  |  |  |  |  |  |
| Databases: Scopus |  |  |  |  |  |  |  |  |  |  |
| Results: 557030 |  |  |  |  |  |  |  |  |  |  |
|  |  |  |  |  |  |  |  |  |  |  |
| Set#: Search 2 |  |  |  |  |  |  |  |  |  |  |
| Searched for: "development" OR "development validation" |  |  |  |  |  |  |  |  |  |  |
| Databases: Scopus |  |  |  |  |  |  |  |  |  |  |
| Results: 11396970 |  |  |  |  |  |  |  |  |  |  |
|  |  |  |  |  |  |  |  |  |  |  |
| Set#: Search 3 |  |  |  |  |  |  |  |  |  |  |
| Searched for: "electronic medical record" OR "electronic health record" |  |  |  |  |  |  |  |  |  |  |
| Databases: Scopus |  |  |  |  |  |  |  |  |  |  |
| Results: 156099 |  |  |  |  |  |  |  |  |  |  |
|  |  |  |  |  |  |  |  |  |  |  |
| Set#: Search 4 |  |  |  |  |  |  |  |  |  |  |
| Searched for: "global trigger tool" OR "automated trigger tool" |  |  |  |  |  |  |  |  |  |  |
| Databases: Scopus |  |  |  |  |  |  |  |  |  |  |
| Results: 945 |  |  |  |  |  |  |  |  |  |  |
|  |  |  |  |  |  |  |  |  |  |  |
| Set#: Search 5 |  |  |  |  |  |  |  |  |  |  |
| Searched for: ("primary care" OR "public health center" OR "ambulatory care" OR "outpatient clinic") AND ("development" OR "development validation") AND ("electronic medical record" OR "electronic health record") AND ("global trigger tool" OR "automated trigger tool") |  |  |  |  |  |  |  |  |  |  |
| Databases: ABI/INFORM Collection, Coronavirus Research Database, Ebook Central, Publicly Available Content Database |  |  |  |  |  |  |  |  |  |  |
| These databases are searched for part of your query. |  |  |  |  |  |  |  |  |  |  |
| Results: 108 |  |  |  |  |  |  |  |  |  |  |
|  |  |  |  |  |  |  |  |  |  |  |
| ---------------------------------------------------------- |  |  |  |  |  |  |  |  |  |  |

1. PROQUEST

| Search Strategy |  |  |  |  |  |  |  |  |  |  |
| --- | --- | --- | --- | --- | --- | --- | --- | --- | --- | --- |
|  |  |  |  |  |  |  |  |  |  |  |
| Set#: Search 1 |  |  |  |  |  |  |  |  |  |  |
| Searched for: "primary care" OR "public health center" OR "ambulatory care" OR "outpatient clinic" |  |  |  |  |  |  |  |  |  |  |
| Databases: ABI/INFORM Collection, Coronavirus Research Database, Ebook Central, Publicly Available Content Database |  |  |  |  |  |  |  |  |  |  |
| Results: 709001 |  |  |  |  |  |  |  |  |  |  |
|  |  |  |  |  |  |  |  |  |  |  |
| Set#: Search 2 |  |  |  |  |  |  |  |  |  |  |
| Searched for: "development" OR "development validation" |  |  |  |  |  |  |  |  |  |  |
| Databases: ABI/INFORM Collection, Coronavirus Research Database, Ebook Central, Publicly Available Content Database |  |  |  |  |  |  |  |  |  |  |
| Results: 42080825 |  |  |  |  |  |  |  |  |  |  |
|  |  |  |  |  |  |  |  |  |  |  |
| Set#: Search 3 |  |  |  |  |  |  |  |  |  |  |
| Searched for: "electronic medical record" OR "electronic health record" |  |  |  |  |  |  |  |  |  |  |
| Databases: ABI/INFORM Collection, Coronavirus Research Database, Ebook Central, Publicly Available Content Database |  |  |  |  |  |  |  |  |  |  |
| Results: 141882 |  |  |  |  |  |  |  |  |  |  |
|  |  |  |  |  |  |  |  |  |  |  |
| Set#: Search 4 |  |  |  |  |  |  |  |  |  |  |
| Searched for: "global trigger tool" OR "automated trigger tool" |  |  |  |  |  |  |  |  |  |  |
| Databases: ABI/INFORM Collection, Coronavirus Research Database, Ebook Central, Publicly Available Content Database |  |  |  |  |  |  |  |  |  |  |
| Results: 319 |  |  |  |  |  |  |  |  |  |  |
|  |  |  |  |  |  |  |  |  |  |  |
| Set#: Search 5 |  |  |  |  |  |  |  |  |  |  |
| Searched for: ("primary care" OR "public health center" OR "ambulatory care" OR "outpatient clinic") AND ("development" OR "development validation") AND ("electronic medical record" OR "electronic health record") AND ("global trigger tool" OR "automated trigger tool") |  |  |  |  |  |  |  |  |  |  |
| Databases: ABI/INFORM Collection, Coronavirus Research Database, Ebook Central, Publicly Available Content Database |  |  |  |  |  |  |  |  |  |  |
| These databases are searched for part of your query. |  |  |  |  |  |  |  |  |  |  |
| Results: 35 |  |  |  |  |  |  |  |  |  |  |
|  |  |  |  |  |  |  |  |  |  |  |
| ---------------------------------------------------------- |  |  |  |  |  |  |  |  |  |  |
